# Feasibility of low dose chest CT for virtual bronchoscopy navigation in a porcine model

**DOI:** 10.1186/s12931-019-1109-8

**Published:** 2019-07-08

**Authors:** Insu Kim, Geewon Lee, Jung Seop Eom, Hyo Yeong Ahn, Ahreum Kim

**Affiliations:** 1 Department of Internal Medicine, Pusan National University School of Medicine, 179 Gudeok-ro, Seo-gu, Busan, 49241 Korea; 2 Department of Radiology, Pusan National University School of Medicine, Busan, Korea; 3 Department of Thoracic and Cardiovascular Surgery, Pusan National University School of Medicine, Busan, Korea; 4 Biostatistics Team of Regional Center for Respiratory Diseases, Pusan National University Hospital, Busan, Korea

**Keywords:** Bronchoscopy, Diagnosis, Lung neoplasms, Multisection computed tomography, Radiation

## Abstract

**Background:**

Virtual bronchoscopy navigation (VBN) is widely used for assistance in the histological examination of lung nodules. However, little is known about the optimal CT radiation dose for VBN. Therefore, we performed an animal study to evaluate the feasibility of low dose CT (LDCT) for VBN.

**Methods:**

Ten pigs underwent standard dose CT (as a reference) and four different LDCT protocols: LDCT 1, 120 kVp, 15 mAs; LDCT 2, 120 kVp, 8 mAs; LDCT 3, 100 kVp, 7 mAs; and LDCT 4, 100 kVp, 4 mAs. As targets for the VBN, 10 mm virtual lesions were created in the central and peripheral bronchi. To assess the performance of the VBN, the navigation direction (direction of reconstructed pathways to the target) and the number of branching’s (the number of peripheral bronchi to the target) were evaluated.

**Results:**

The mean effective doses significantly differed across the four LDCTs (*P* <  0.001). For both central and peripheral virtual targets, there were significant differences in the accuracy of the navigation direction and the number of branching’s of the VBNs across the four LDCTs (*P* <  0.001 for all). Regarding the accuracy of the navigation direction and the number of branching’s, the areas under the curves of the ROCs were 0.9352 and 0.9324, respectively, for central virtual targets, and 0.8696 and 0.8783, respectively, for peripheral virtual targets. Youden’s index indicated that the optimal effective CT scan dose for both central and peripheral virtual targets was 0.238 mSv.

**Conclusions:**

LDCT is feasible for VBN.

## Background

According to the National Lung Screening Trial, low dose computed tomography (LDCT) screening in high-risk individuals can reduce lung cancer mortality by 20% compared with screening using chest radiographs [1]. However, there are still major limitations with the use of LDCT screening, such as the low prevalence of lung cancer and high rate of false positive results [2, 3]. In particular, most lung nodules detected on LDCT screening are benign, and histological examination should therefore be included in the workup for the confirmative diagnosis of lung cancer [4].

Fortunately, recent developments in guided bronchoscopy allow a minimally invasive approach for histological sampling of small lung nodules with an acceptable diagnostic yield [5–7]. Virtual bronchoscopy navigation (VBN) uses continuous volume CT data to provide three-dimensional virtual images of the bronchial pathway to a peripheral lesion [8, 9]. Previous studies demonstrated that a multimodal approach combining radial probe endobronchial ultrasound with VBN provided 70–90% successful diagnosis of peripheral lung lesions, with low complication rates of 1–3% [10, 11].

However, VBN requires thin-section volumetric CT scans, and accordingly, patients with peripheral lung nodules on LDCT screening are required to undergo an additional high-radiation-dose CT scan. If the VBN system was able to function appropriately using the existing LDCT data, patients with a lung nodule could avoid unnecessary additional radiation exposure. Therefore, in the present study, we evaluated the feasibility of LDCT for VBN through an animal study using live porcine models.

## Methods

### Animal models

Ten live female farm pigs were used in the present study. At the time of the CT scan, the mean age was 8 weeks (range, 10–12 weeks) and the mean weight was 41 kg (range, 34–46 kg). The animal care and use protocol of the present study was reviewed and approved by the Institutional Animal Care and Use Committee of Pusan National University Yangsan Hospital (Approval No. 2018–058).

### CT protocol and acquisition

**Fig. 1:**
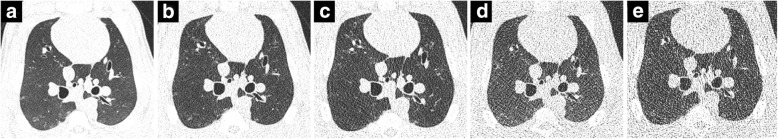
Representative images of standard and low dose CT scans. **a** Standard dose CT. **b**-**e** Low dose CT scans 1–4

The ten pigs each underwent five consecutive CT scans consisting of four LDCTs with different kVp and mAs settings, and a standard dose CT as a reference. All CT images were obtained on a multidetector scanner (Siemens Somatom Definition AS+; Siemens Healthcare, Forchheim, Germany) using a helical scan technique without a change in the position of the pigs or a time delay between consecutive scans. To acquire the CT scans at total lung capacity, a bag valve mask was used to provide positive pressure ventilation to the pigs under conditions of general anesthesia, which were attained using an intramuscular injection of alfaxalone (5 mg/kg) and an intravenous injection of vecuronium (0.1 mg/kg). No intravenous contrast medium was injected, and the detailed CT protocols are presented in Table 1. First, the standard dose CT was performed at 120 kVp and 50 mAs, which was then followed by the four low dose CT scans at 120 kVp and 15 mAs (LDCT protocol 1), 120 kVp and 8 mAs (LDCT protocol 2), 100 kVp and 7 mAs (LDCT protocol 3), and 100 kVp and 4 mAs (LDCT protocol 4). The rotation time was 0.28 s for all CT scans, and all images were reconstructed as 0.6 mm axial scans using both a high-spatial-resolution kernel (b50f) and a soft-resolution kernel (b31f). All CT scans were saved on a picture archiving and communication system (PACS), which connected with the VBN system (LungPoint; Broncus Medical, Mountain View, CA) via the PACS network. Representative CT scans are shown in Fig. 1.

**Table 1:** CT protocols

	Standard dose CT	LDCT protocol 1	LDCT protocol 2	LDCT protocol 3	LDCT protocol 4
Peak kilovoltage, kVp	120	120	120	100	100
Milliamperes, mAs	50	15	8	7	4

*LDCT* low dose computed tomography

To estimate the radiation dose, the dose-length products were recorded from the scanner output reports and multiplied by a conversion coefficient (k = 0.014 mSv/mGy∙cm^2^ for the adult chest) to obtain the effective dose [12].

### VBN and virtual targets

**Fig. 2:**
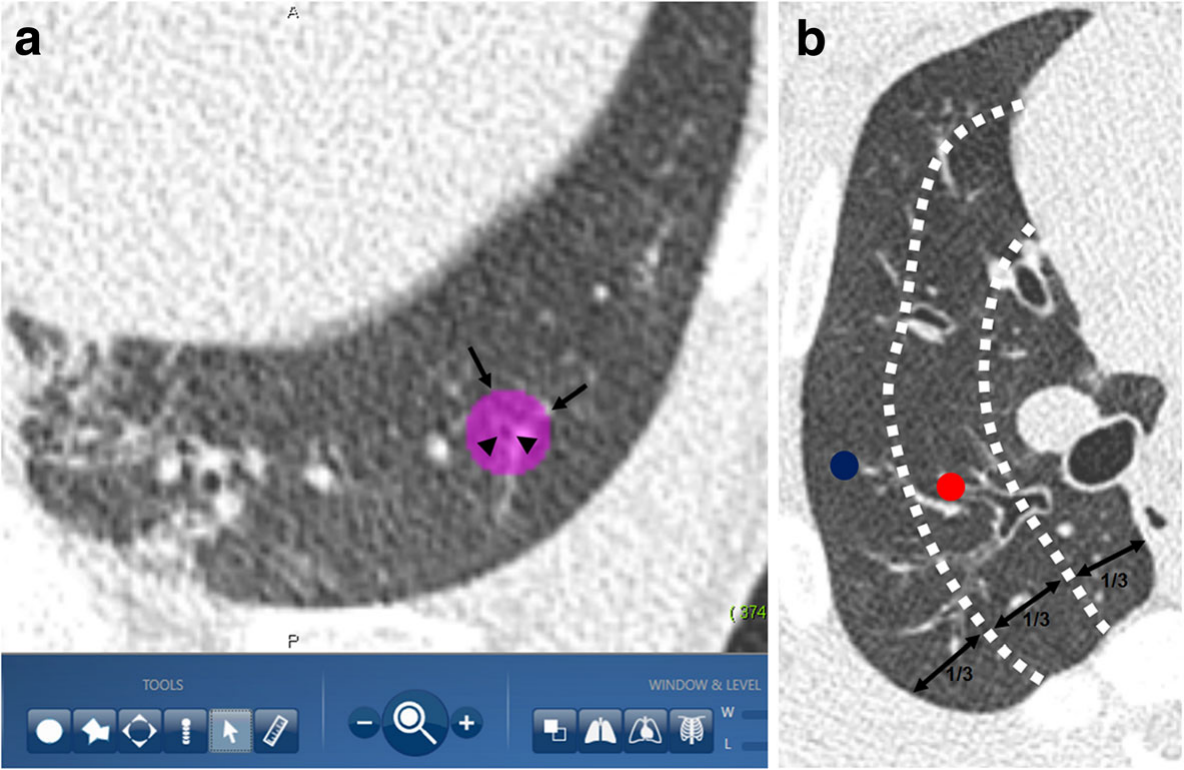
Virtual targets for the virtual bronchoscopy navigation. **a** Creation of a three-dimensional virtual target (arrow) located on the peripheral bronchus (arrowhead) using LungPoint software. **b** Central (red circle) and peripheral (blue circle) virtual targets created in the middle and outer one-third of the lung parenchyma, respectively

Once the CT data (one standard CT and the four LDCT protocols) had been directly imported into the VBN system across our network, LungPoint software was used to automatically analyse the volumetric CT images. To perform the VBN, two types of virtual targets (one peripheral and one central target) were created in each of the six lobes of the porcine models using the LungPoint software to place a three-dimensional spherical marker (10 mm in diameter and 524 mm^3^ in volume) on the appropriate CT images (Fig. 2a). All virtual targets were located beyond the point where the bronchus had a diameter of less than 5 mm. In detail, the axial CT images were divided into three zones (Fig. 2b), and the targets in the middle and outer one-third of the lung parenchyma were defined as central and peripheral virtual targets, respectively. Finally, 12 virtual targets (six central and six peripheral) were created in each porcine model. Once the virtual targets were created, the LungPoint software displayed the three-dimensional virtual bronchial pathways, with these being automatically calculated and reconstructed to reach points as close as possible to the virtual targets.

### Performance of the VBN

**Fig. 3:**
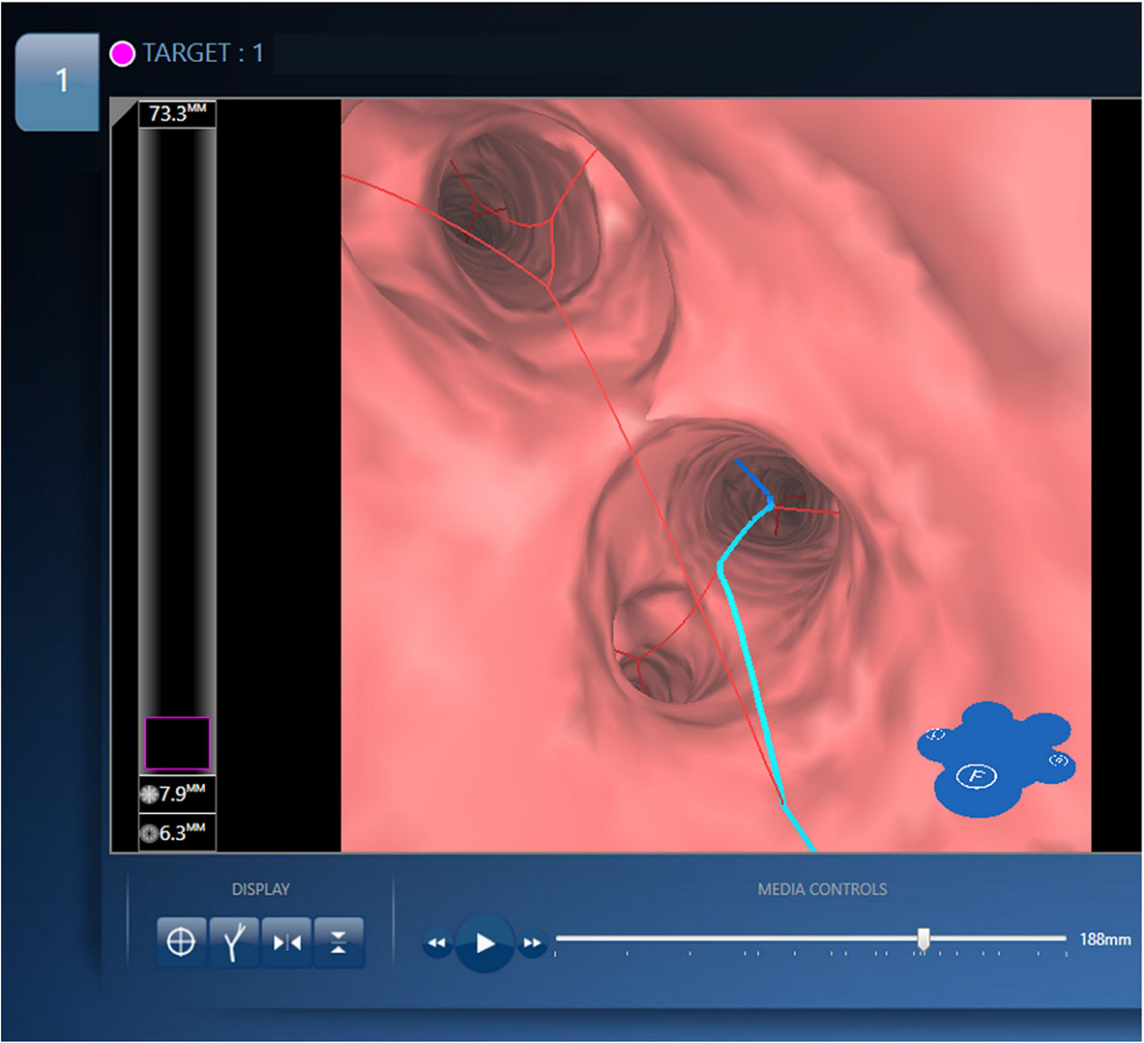
Guidance screen of the VBN. The red line represents all bronchial pathways and the blue line is the automatically searched-for route with the closest access to the virtual target. The navigation direction accuracy was defined as the concordance in the progression of the blue line between the VBN using low dose CT and that using standard dose CT. The number of branching’s was defined as the number of red lines that directly branched from the blue line, and the accuracy of the number of branching’s was defined according to the concordance of this number between the VBNs using low dose and standard dose CT. VBN = virtual bronchoscopy navigation

The LungPoint software provides up to three reconstructed paths, with the first path generally being the most accurate in comparison with the second and third paths. In each porcine model, the first paths reconstructed by the LungPoint system using each of the four LDCT protocols were compared with that reconstructed using the standard reference CT. Two variables of the VBN system were investigated to compare the performance of the VBN using the different-dose CT acquisitions: 1) the navigation direction, which was defined as the direction of the reconstructed bronchial pathways to the virtual target, and 2) the number of branching’s, which was defined as the number of bronchial branches along the path to the virtual targets after the lobar bronchus (please see Fig. 3 for an illustration of how the accuracy measurements were obtained).

### Statistical analysis

All continuous variables are presented as the mean (range). Categorical variables were compared using Pearson’s chi-squared test. The Kruskal-Wallis test was used to compare the effective dose across the four LDCT protocols. Receiver operating characteristic (ROC) curves and the area under the curve (AUC) of the ROCs were calculated to evaluate the feasibility of LDCT for VBN in terms of radiation dose and VBN accuracy. Youden’s index was used to determine the optimal CT protocol with low radiation and acceptable VBN accuracy [13]. A two-sided *P*-value of < 0.05 was considered to indicate statistical significance, and SAS version 9.4 (SAS Institute, Cary, NC) was used for the statistical analyses.

## Results

A total of 120 virtual targets (60 central and 60 peripheral virtual targets) were created using LungPoint software. The mean bronchial diameters at the positions where the central and peripheral virtual targets were placed were 2.0 mm (1.2–4.6) and 1.5 mm (0.8–2.3), respectively. The mean distances from the pleura to the central and peripheral virtual targets were 32.7 (8.5–59.3) and 8.4 mm (1.1–15.4), respectively. The mean numbers of branching’s to the central and peripheral virtual targets were 4.7 (3–10) and 9 (5–23), respectively.

### Effective doses

The mean effective dose of the reference standard CT was 1.68 mSv (1.47–2.03). There were significant differences in the mean effective doses across the four LDCT protocols (0.51, 0.28, 0.15, and 0.09 mSv for LDCTs 1–4, respectively; *P* <  0.001).

### Accuracy of navigation direction

For the central virtual targets, the navigation direction accuracy of the VBN using both the LDCT 1 and 2 acquisitions was 100% in comparison with that using the standard CT (Table 2). However, the VBN navigation direction accuracies using LDCT 3 and 4 were 81.7 and 43.3%, respectively. For the central virtual targets, there were significant differences in the navigation direction accuracies across the four LDCT protocols (*P* <  0.001).

**Table 2:** Comparisons of the VBN performance using the four LDCTs

	LDCT 1	LDCT 2	LDCT 3	LDCT 4	*P* value
Accuracy of navigation direction					
Central targets, %	100	100	81.7	43.3	< 0.001
Peripheral targets, %	100	100	90	31.7	< 0.001
Accuracy of the number of branching’s					
Central targets, %	100	85	45	0	< 0.001
Peripheral targets, %	85	51.7	21.7	0	< 0.001

*VBN* virtual bronchoscopy navigation, *LDCT* low dose computed tomography

For peripheral virtual targets, the navigation direction accuracies were also 100% for LDCT 1 and 2, whereas they were 90 and 31.7% for LDCT 3 and 4, respectively. The accuracies of the navigation direction to peripheral virtual targets showed significant differences across the four LDCT protocols (*P* <  0.001).

### Accuracy of the number of branching’s

In comparison with the standard dose CT, the number of branching’s to the central virtual targets had accuracies of 100, 85, 45, and 0% for LDCTs 1–4, respectively (*P* <  0.001). For the peripheral virtual targets, these accuracy values were 85, 51.7, 21.7, and 0% for LDCTs 1–4, respectively (*P* <  0.001).

### Optimal effective dose

**Fig. 4:**
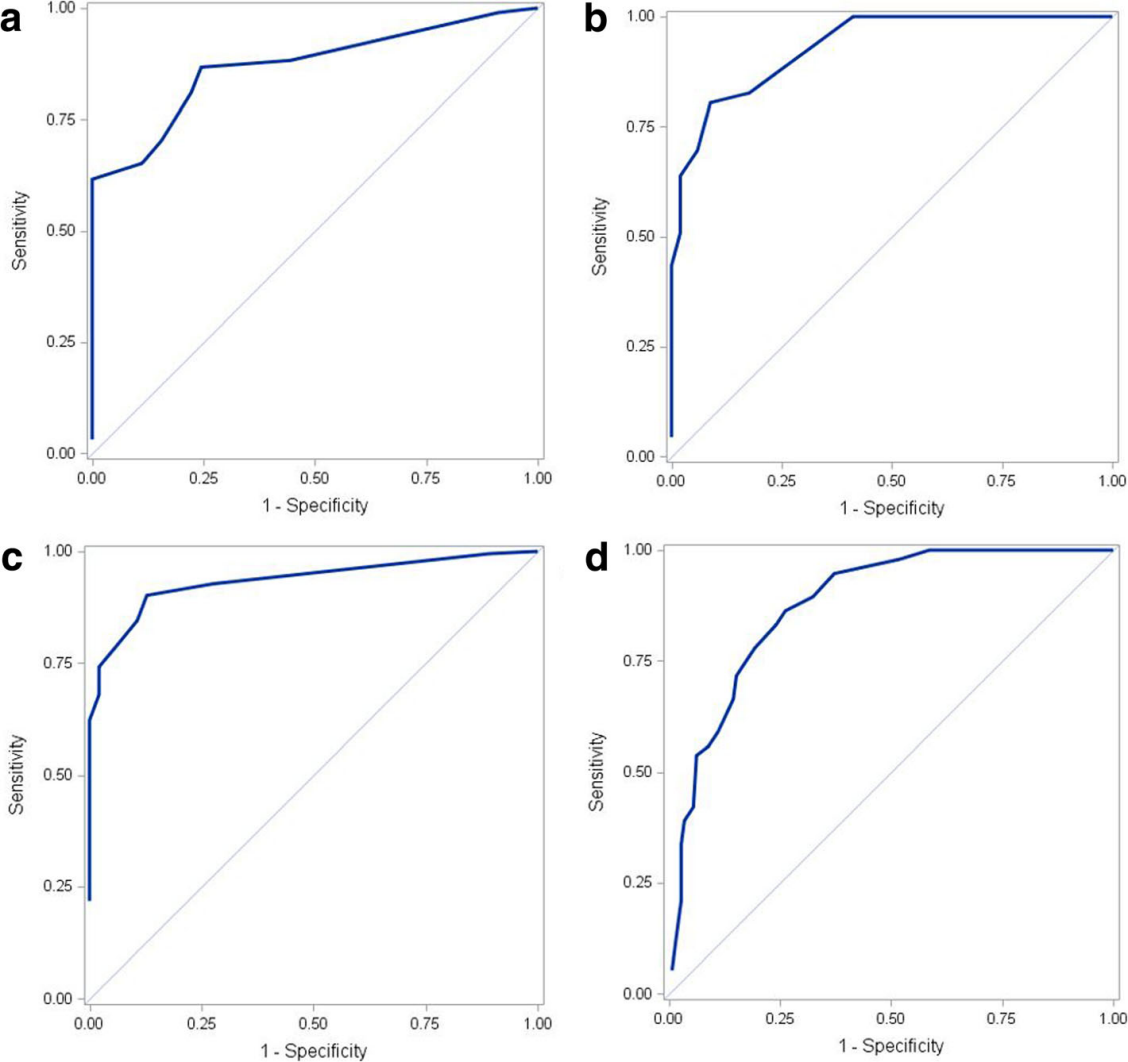
ROC curves of the optimal effective low dose CT scan doses for VBN performance. **a** ROC curve of the navigation direction for central virtual targets. **b** ROC curve of the number of branching’s for central virtual targets. **c** ROC curve of the navigation direction for peripheral virtual targets. **d** ROC curve of the number of branching’s for peripheral virtual targets. VBN = virtual bronchoscopy navigation

The ROC curves based on the effective doses are shown in Fig. 4. For the central virtual targets, the AUCs of the accuracy values of the navigation direction and the number of branching’s were 0.8696 and 0.9324, respectively (Table 3), while for peripheral virtual targets these values were 0.9352 and 0.8783, respectively. Analysis of the Youden’s index indicated that the optimal effective doses for central virtual targets were 0.126 and 0.238 mSv for the navigation direction and number of branching’s, respectively. For the peripheral virtual targets, the optimal effective doses were also 0.126 and 0.238 mSv for the navigation direction and number of branching’s, respectively.

**Table 3:** Analysis of the optimal effective dose for VBN

	Central virtual targets	Peripheral virtual targets
AUC of ROC curve based on effective dose		
Navigation direction	0.8696	0.9352
Number of branching’s	0.9324	0.8783
Youden’s index, mSv		
Navigation direction	0.126	0.126
Number of branching’s	0.238	0.238

*VBN* virtual bronchoscopy navigation, *AUC* area under the curve, *ROC* receiver operating characteristic

## Discussion

Using VBN for three-dimensional guidance, bronchoscopy can approach a peripheral lung lesion through the complicated bronchial tree [14]. Although previous studies reported VBN as a promising navigational modality [15, 16], they did not evaluate the optimal CT protocol for VBN performance. The results of our animal experiment suggest that VBN can be performed using LDCT data. As far as we are aware, this is the first report in which the optimal effective dose CT scan for VBN was evaluated.

Because the individuals who undergo screening for a high-risk of lung cancer typically have a longstanding smoking history that leads to pulmonary emphysema and reduced lung functions [17, 18], guided bronchoscopy combined with a navigational modality such as VBN for the histological examination of peripheral lung lesions is becoming increasingly important [16, 19, 20]. A previous randomised trial reported that when radial probe endobronchial ultrasound was combined with VBN, the diagnostic yield of peripheral lung lesions was improved in comparison with procedures using only radial probe endobronchial ultrasound [10]. In addition, VBN requires no specific training, and the overall complication rate of bronchoscopy with VBN is reported to be only 1% (95% confidence interval, 0.2–1.8%) [9].

As the accuracy of VBN depends on the quality of the acquired CT information, the reconstruction of CT images with a slice thickness of 1 mm or less is generally recommended for the various VBN systems [9, 14]. When a peripheral lung lesion is found on LDCT screening or the raw data from a previous conventional CT scan are unavailable, another CT scan with an additional radiation exposure is generally performed to allow the reconstruction of the thin sections required for VBN. If a peripheral lung lesion is revealed to be benign on pathological examination, the patient may have received an unnecessary extra CT scan for the VBN, and the radiation hazard from the unnecessary CT could consequently increase the future risk of cancer development [21, 22]. Thus, in patients undergoing repeated CT scans, the ALARA (as low as reasonably achievable) principle is relevant [23], and in this context, our study is unique and valuable in trying to find the optimal CT protocol for VBN in respect to the radiation dose to the patient.

For central virtual targets, the accuracy of VBN using LDCT 1 or 2 was identical to that using standard dose CT. When the accuracies of both the navigation direction and the number of branching’s are considered, the optimal effective CT scan dose for central virtual targets was 0.238 mSv, which corresponds with LDCT protocol 2 (mean effective dose = 0.28 mSv). Although the accuracies of VBN for peripheral virtual targets using LDCT were lower than those for central virtual targets, the optimal effective CT scan dose was again calculated as 0.238 mSv using Youden’s index, which also corresponded with LDCT protocol 2. Our results suggest that VBN can be performed with LDCT data with a minimum effective dose of 0.238 mSv. If VBN can be accurately performed using LDCT data, the LDCT data from lung cancer screening could be used for the VBN, without the need for an additional CT scan. Even if the raw CT scan data are unavailable, an additional CT scan for VBN purposes could be performed using a low dose protocol.

The present study has several limitations requiring acknowledgement. First, it is uncertain whether the CT scans were performed at total lung capacity in each porcine model. However, we used a bag valve mask during the CT scan, which provided positive pressure to the peripheral bronchi of the pigs. Second, the effective doses were calculated with a conversion coefficient for the adult chest (k = 0.014 mSv/mGy∙cm^2^). Although the porcine lung may not be a perfect substitute for adult human lungs, this is a widely used simplified method to quickly estimate the effective dose from the dose-length product and sets of age- and body region-specific k-coefficients [23]. Third, because all the VBNs were performed using LungPoint software, our results cannot be generalised to other VBN systems. Fourth, we used experimental pigs with biased sex and weight. Finally, although the anatomical structure of the respiratory system is similar between humans and pigs [24], the results of our animal experiments should be verified in a future human study using novel technology [25, 26].

## Conclusions

The results of this study using live porcine models suggest that VBN can be performed using LDCT data. The minimum required effective dose for VBN operation is 0.238 mSv, which corresponds with the LDCT protocol 2 of the present study (peak kilovoltage, 120 kVp; milliamperes, 8 mAs).

## Data Availability

Please contact author for data requests.

## Abbreviations

ALARA: As low as reasonably achievable
AUC: Area under the curve
LDCT: Low dose computed tomography
PACS: Picture archiving and communication system
ROC: Receiver operating characteristic
VBN: Virtual bronchoscopy navigation
